# Successful Allogeneic Hematopoietic Stem Cell Transplantation of a Patient Suffering from Type II Congenital Dyserythropoietic Anemia A Rare Case Report from Western India

**DOI:** 10.1155/2015/792485

**Published:** 2015-01-26

**Authors:** Gaurang Modi, Sandip Shah, Irappa Madabhavi, Harsha Panchal, Apurva Patel, Urmila Uparkar, Asha Anand, Sonia Parikh, Kinnari Patel, Kamlesh Shah, Swaroop Revannasiddaiah

**Affiliations:** ^1^Department of Medical and Paediatric Oncology, GCRI, Ahmedabad, Gujarat 380016, India; ^2^Department of Radiotherapy, Government Medical College, Haldwani, Uttarakhand 263139, India

## Abstract

The most frequent form of congenital dyserythropoiesis (CDA) is congenital dyserythropoietic anemia II (CDA II). CDA II is a rare genetic anemia in humans, inherited in an autosomally recessive mode, characterized by hepatosplenomegaly normocytic anemia and hemolytic jaundice. Patients are usually transfusion-independent except in severe type. We are here reporting a case of severe transfusion-dependent type II congenital dyserythropoietic anemia in a 5-year-old patient who has undergone allogeneic hematopoietic stem cell transplantation (HSCT) at our bone marrow transplantation centre. Patient has had up until now more than 14 mL/kg/month of packed cell volume (PCV), which he required every 15 to 20 days to maintain his hemoglobin of 10 gm/dL and hematocrit of 30%. His pre-HSCT serum ferritin was 1500 ng/mL and he was on iron chelating therapy. Donor was HLA identical sibling (younger brother). The preparative regimen used was busulfan, cyclophosphamide, and antithymocyte globulin (Thymoglobulin). Cyclosporine and short-term methotrexate were used for graft versus host disease (GVHD) prophylaxis. Engraftment of donor cells was quick and the posttransplant course was uneventful. The patient is presently alive and doing well and he has been transfusion-independent for the past 33 months after HSCT.

## 1. Introduction

Congenital dyserythropoietic anemia (CDA) is a rare blood disorder, similar to the thalassemias. CDA is one of many types of anemia, characterized by ineffective erythropoiesis, resulting from a decrease in the number of red blood cells (RBCs) in the body and a less than normal quantity of hemoglobin in the blood. Traditionally, CDA have been classified into 3 major types (CDA I, CDA II, and CDA III); recently, additional variants have been described. CDA type II (CDA II) is the most frequent type of congenital dyserythropoietic anemias.

Congenital dyserythropoietic anemia type II is a genetic hyporegenerative anemia characterized by ineffective erythropoiesis and distinct morphological abnormalities of the erythroblasts in the bone marrow (BM). Type II congenital dyserythropoietic anemia (CDA II) is an autosomal recessive disorder characterized by hemolytic jaundice, mild to moderate hepatosplenomegaly, and normocytic anaemia [1, 2]. Most of the patients are transfusion-independent except for 10 to 20% of the cases, who are transfusion-dependent; this could account for the severity of the clinical outcome [3, 4]. Management usually includes blood transfusion, iron chelating therapy, and splenectomy. Transfusion-dependent patients usually require allogeneic HSCT (hematopoietic stem cell transplantation) from HLA identical donor. Only few published case reports of allogeneic HSCT in CDA patients are available.

## 2. Case Report

Child was apparently asymptomatic till the age of 3 years. He complained of lethargy and weakness for one to two months prior to presentation. His routine investigations showed hemoglobin (Hb) of 5 gm/dL, indirect hyperbilirubinemia, and mild-moderate hepatosplenomegaly. He had unremarkable birth and family history. His peripheral smear was showing severe microcytic hypochromic anemia with anisocytosis, poikilocytosis, and low MCV. After ruling out hemolytic causes of anemia including thalassemia, sickling disease, and G6PD deficiency, patient underwent marrow aspiration and biopsy. It showed erythroid hyperplasia and feature of dyserythropoiesis such as binucleation (20%), multinucleation, and karyorrhexis. The other cell lines were normal. The genetic analysis of the patient's peripheral blood revealed SEC23 B gene mutation by real-time polymerase chain reaction (RT-PCR). In view of marrow picture and genetic mutational analysis, final diagnosis of CDA II was made. At the age of 5 years, he was referred to our hematology unit for further management. Patient has had up until now more than 14 mL/kg/month of packed cell volume (PCV).

On examination, he had marked pallor, with stable vitals. Abdominal examination revealed palpable splenomegaly of 4 cm below the left costal margin and hepatomegaly of 5 cm below the right costal margin. His pre-HSCT serum ferritin was 1500 ng/mL; he was on regular iron chelating therapy (deferasirox 30 mg/kg) once a day. His renal function tests and liver function tests were within normal range except for mild indirect hyperbilirubinemia. Other therapeutic options such as splenectomy were explained to patient's relatives. Family was reluctant to go for splenectomy. Liver biopsy revealed moderate iron overload and no fibrosis was seen.

Allogeneic HSCT (hematopoietic stem cell transplantation) from his HLA-matched sibling was planned for patient. The donor was a 2-year-old younger brother, in whom CDA II was ruled out. The patient had 10/10 HLA match by low resolution assay. Cytomegalovirus (CMV) status of both the donor and recipient was negative. Graft type was of peripheral blood stem cells (PBSC). Both patient and donor blood group were AB positive. Pre-HSCT work-up of the patient was normal. After taking written consent regarding HSCT, double lumen Hickman catheter insertion was done by expert anesthetist.

Initially, 45 days prior to the start of conditioning therapy, bone marrow suppression was done with azathioprine and hypertransfusion of packed cell volume to maintain the Hb and hematocrit of 15 gm/dL and 40–45%, respectively. Myeloablative conditioning regimen of busulfan 0.8 mg/kg/dose intravenous for 16 doses (−Day 10 to –Day 7), cyclophosphamide intravenous 50 mg/kg/day with mesna (−Day 5 to −Day 2), and antithymocyte globulin (Thymoglobulin) intravenous 1.5 mg/kg/day (−Day 3 to –Day 1) was used. Ursodeoxycholic acid 12 mg/kg/day was given orally from the day before starting the conditioning regimen until +Day 90 after transplant to prevent the venoocclusive disease. Oral cyclosporine 5 mg/kg was started from –Day 1 and continued for +Day 60 to prevent graft versus host disease (GVHD). On Day 0, stem cell infusion was done with dose of CD34+ cells of 7 × 10^6^ per kg weight. IV methotrexate was given on +Day 1, +Day 3, +Day 6, and +Day 11 as graft versus host disease (GVHD) prophylaxis. Initially supportive measures were taken to correct low hemoglobin and platelet count. Engraftment for neutrophils was achieved on +Day 14 with 3 consecutive absolute neutrophils counts of more than 500 cumm/dL. Engraftment for platelets was achieved on +Day 19 with 3 consecutive platelet counts of more than 50000 cumm/dL without any component support. 100% donor chimerism for both myeloid and lymphoid cells was achieved by +Day 45.

Patient was given prophylactic antibacterial, antifungal, and anti-*P. jirovecii* treatment during HSCT procedure. Engraftment of donor cells was prompt and the post-HSCT duration was uneventful. No acute or chronic graft versus host disease (GVHD) was noted. Cyclosporine and acyclovir were continued for 6 and 12 months, respectively. Post-HSCT serial complete blood counts and hemoglobin of patient were normalized on +Day 40 and he was never given PCV (packed cell volume) or platelets since then. Thirty-three months after HSCT, the patient is alive with normal hemoglobin level and is not receiving any immune suppressive therapy at present. Iron chelating therapy was stopped 3 months after transplant, when the serum ferritin level was noted to be less than 1000 ng/mL. Now, he is almost 8 years old, under regular surveillance at our transplant centre without any symptoms.

## 3. Discussion

The congenital dyserythropoietic anemia (CDA) comprises a group of rare hereditary disorders, characterized by ineffective erythropoiesis as the predominant mechanism of anemia and by distinct morphological abnormalities of the majority of erythroblasts in the bone marrow. The term was first used by Crookston et al. [11]. The diagnosis should be suspected in any case of congenital anemia with features of hemolytic anemia. Various types of CDA are noted of which the most frequent form is type II. Approximately 370 cases of CDAII are reported till date. Although this disorder is considered to be congenital, it is interesting that this type of anemia can be diagnosed in all age groups. The molecular defects in the gene SEC23B (loci is 20p11.23) are the confirmatory criteria for the diagnosis of the CDA II [12]. Erythrocytes of CDA II patients lyse in acidified serum (Ham test) because of an IgM class antibody that recognizes an antigen present on CDA II cells but absent on normal cells. So the acronym HEMPAS (hemolytic anemia with a positive acidified serum test) was commonly used as a synonym for CDA II [13].

Only 10–20% of the patients have severe anemia; in that case, management focuses mainly on supportive care with the use of transfusions, iron chelating therapy, allogeneic HSCT, and rarely splenectomy. Iron accumulates steadily throughout life, with kinetics similar to untreated hereditary hemochromatosis [14], and at present management of iron overload should follow guidelines for thalassemia. Parents of our patient did not want lifelong blood transfusion, regular iron chelating agents, or risk of infection susceptibility after splenectomy. HSCT was the only curative option to avoid the above complications.

Curative therapy for CDA focuses on the use of HSCT using fully matched sibling donors [8]. Some of the CDA II cases which have been managed successfully with HSCT with excellent results were mentioned in the literature. It has been reported to be curative in a few severe cases of CDA including transfusion-dependent CDA type I, CDA type II, Ham test-negative CDA type II, and an unclassified CDA (Table 1). HSCT was successful in some children with exceptionally severe anemia [15]. So there is a paucity of literature available to guide us through the application of HSCT in this case. In many instances, a matched sibling donor is not available and conventional conditioning may be poorly tolerated in many patients. Lucarelli et al. described risk stratification of thalassemia patients which is defined by the following risk factors: (1) hepatomegaly >2 cm, (2) portal fibrosis of any degree, and (3) inadequate compliance with the chelating agents. Class 1 means no risk factors, Class 2 had one or two risk factors, and Class 3 included all 3 risk factors. We had assigned our patient to Class 2 status (in view of presence of hepatomegaly) in which HSCT has also been reported as the only viable curative option [16].

In developing countries like SEAR regions with financial constraints and limited resources, CDA II patients can be benefited best with the use of myeloablative conditioning regimen, infusion of high stem cell dose, and all the measures which would lead to reduced infection rate and duration of hospital stay. We have demonstrated a successful and safe application of aggressive iron chelating agents before HSCT followed by successful HSCT.

## 4. Conclusion

Due to rarity of disease, no definitive treatment guidelines are available but allogeneic HSCT should be considered in severe cases. As in thalassemia early and regular iron chelating agents in CDA before HSCT give better results. In the developing world with limited resources and financial constraint, severe case of CDA can be managed successfully with allogeneic HSCT by using myeloablative conditioning regimen, infusing high CD34+ stem cell dose, to ensure early engraftment; appropriate and adequate GVHD measure would contribute to reducing infection rate and duration of hospital stay.

## Highlights: Learning Points

- The congenital dyserythropoietic anemia (CDA) is a group of rare hereditary disorders, characterized by ineffective erythropoiesis and specific morphologic abnormalities of erythroblasts in the bone marrow.
- Traditionally, CDA have been classified into 3 major types (CDA I, CDA II, and CDA III); recently, additional variants have been described.
- The diagnosis of CDA should be suspected in any case of congenital anemia with features of hemolytic anemia.
- The molecular defects in the gene SEC23B (loci is 20p11.23) are the cause of CDAII.
- There is a paucity of literature available to guide the management of CDA.
- Allogeneic HSCT is the only curative strategy available in severe transfusion-dependent CDA patients.

## Figures and Tables

**Table 1:** HSCT done in various types of CDA.

Year	Author	Type of CDA
1996	Ariffin et al. [5]	Type not documented

2012	Iolascon et al. [3]	CDA II with thalassemia trait

2002	Ayas et al. [6]	CDA I

2002	Remacha et al. [7]	CDA II with a negative Ham test

2012	Buchbinder et al. [8]	Unrelated hematopoietic stem cell transplantation in a patient with congenital dyserythropoietic anemia (CDA) and iron overload

2014	Russo et al. [9]	Successful hematopoietic stem cell transplantation in a patient with CDA II

2014	Braun et al. [10]	Successful treatment of an infant with CDA II by intrauterine transfusions and postnatal stem cell transplantation
